# Characterization of Early Lesions of Human Post-Primary Tuberculosis and Its Progression to Necrosis Using Archival Material of the Pre-Antibiotic Era

**DOI:** 10.3390/pathogens14030224

**Published:** 2025-02-25

**Authors:** Syeda Mariam Riaz, Kurt Hanevik, Lisbet Sviland, Tehmina Mustafa

**Affiliations:** 1 Centre for International Health, Department of Global Public Health and Primary Care, Faculty of Medicine and Dentistry, University of Bergen, 5020 Bergen, Norway; mariamriaz73@gmail.com; 2 Department of Clinical Science, Faculty of Medicine and Dentistry, University of Bergen, 5020 Bergen, Norway; kurt.hanevik@uib.no; 3 National Center for Tropical Infectious Diseases, Medical Department, Haukeland University Hospital, 5020 Bergen, Norway; 4 Department of Clinical Medicine, Faculty of Medicine and Dentistry, University of Bergen, 5020 Bergen, Norway; lisbet.sviland@helse-bergen.no; 5 Department of Pathology, Haukeland University Hospital, 5020 Bergen, Norway; 6 Department of Thoracic Medicine, Haukeland University Hospital, 5020 Bergen, Norway

**Keywords:** post-primary tuberculosis, tuberculosis pneumonia, early lesion, necrotic lesion, macrophages, T cells, programmed death-ligand 1, programmed death

## Abstract

Primary and post-primary TB are distinct entities. Primary TB occurs when the patient is infected with Mycobacterium tuberculosis (MTB) for the first time without prior immunity, and post-primary TB occurs when the patient has developed immunity against the primary infection. Post-primary TB occurs only in humans. It accounts for 80% of all clinical cases and nearly 100% of transmissions of infection. Early lesions of post-primary TB are reversible, and studying it using modern immunological tools holds the key to developing preventive or treatment strategies. Human lung tissue from untreated TB patients was acquired from pathology archives stored at the Gades Institute of Pathology, Haukeland University Hospital, Bergen, Norway, from 1931 to 1947. Manual immunohistochemistry was performed for macrophage (CD68, CD64 and CD163), T cells (CD3 and CD8), matrix metalloproteinases (MMP-9), and markers for programmed death-pathway PD/PDL-1. Digital quantification was performed using Qupath software. In early lesions of post-primary TB, macrophages showed mixed-phenotype M1 and M2, expressed PDL-1, and were compartmentalized in the alveolar space. T-cells expressed PD-1 and were compartmentalized in the interstitial wall surrounding early lesions. MTB antigens and MMP-9 were also found in early lesions. As the lesion progressed towards necrosis, macrophages showed predominant M1 morphology, and expressions of PDL-1, PD-1, CD8^+^ cells, and MTB antigens increased. In the early lesions of post-primary TB, the compartmentalization of macrophages in the alveoli and T cells in the interstitium was shown. The PDL-PD1 pathway probably facilitated the mycobacterial growth by evading host immunity.

## 1. Introduction

Primary tuberculosis (TB) and post-primary TB are two different disease manifestations caused by the same organism, *Mycobacterium tuberculosis* (MTB). This fact was well studied and recognized by the scientific community in the pre-antibiotic era [1]. Primary TB develops after the first exposure to MTB infection. It occurs mainly in children and immunocompromised patients, but is frequently also seen in adults in low-endemic settings. The characteristic lesion of primary TB is the development of a granuloma accompanied by the enlargement and caseation of draining lymph nodes [2]. We have many animal models of primary TB and, thereby, a relatively good understanding of the development and functions of its characteristic lesion, the caseating granuloma. However, no animals produce the post-primary TB lesions that mediate transmission to new hosts [3,4], and there is a scarcity of information on the immunopathology of post-primary TB. Primary TB can also occur as extrapulmonary and miliary TB, resulting from the lymphatic and hematogenous spread of MTB to other organs [5].

Post-primary TB occurs when the individual has already developed immunity against the primary infection. It accounts for 80% of all clinical cases and nearly 100% of transmissions of infection [6]. Post-primary TB is different from primary TB from its inception and starts as an early lesion [7,8,9]. The early lesion of post-primary TB has been studied extensively in humans both pathologically and radiographically in the pre-antibiotic era under different names, including Assmann's focus, early bronchogenic TB or early infiltrate [8,10]. However, the cell subtypes of these lesions and the presence of MTB antigens have yet to be studied with modern immunology tools. An early lesion is characterized by the accumulation of foamy macrophages within the alveolar space [11]. These lesions do not give rise to abnormal physical signs or cause local or constitutional symptoms. These lesions are lobular pneumonia that spread through the bronchi, not through the blood or lymphatics, as does primary TB [2]. It may regress or progress by undergoing necrosis to become caseous pneumonia, which softens and fragments to produce cavities, or be retained to become the focus of fibrocaseous disease, post-primary granuloma [12]. Post-primary granulomas are different from primary TB granulomas and are characterized by the development of necrosis first, which is then contained by granulomatous inflammation. Lesions of post-primary TB develop independently from each other, and lesions in different stages of development can be found compartmentalized within the same lung [9,13]. 

Recent insights into primary TB granuloma biology suggest several new strategies to prevent and treat TB [14]. However, the targetable pathways discovered by studying granulomas in primary TB will not necessarily translate to the setting of post-primary TB, as the pathogenesis of post-primary TB is quite distinct from primary TB and the pulmonary lesions can differ in their cellular organization; this is a prerequisite for insights into potential targetable pathways in post-primary TB, an in-depth understanding of its underlying immunological mechanisms.

Human peripheral blood and bronchoalveolar lavage, occasional lymph node, or other biopsies and lung resections of treated lesions have been used to study the pathogenesis of post-primary TB using modern immunology tools [11]. Unfortunately, none of these tissues contain the early lesions of post-primary disease. Early lesions of post-primary TB can regress and resorb without any symptoms [1,7,10,15]. The reversible nature of these lesions holds the key to developing the preventive or treatment strategies which can make such lesions regress. The main obstacle is the non-availability of human lung tissue with post-primary TB without any interference with antibiotic treatment, thus containing early lesions.

We hypothesize that the foamy macrophages of early lesions of post-primary TB may provide a niche for MTB proliferation rather than controlling the infection. These macrophages accumulate mycobacterial antigens, rendering them ineffective in controlling MTB proliferation by modulating their anti-mycobactericidal mechanisms [16,17]. As seen in many cancers [18], these macrophages might use programmed death-ligand (PDL) overexpression, leading to the deactivation of T cells through the PDL-PD1 pathway or remove T-cells by inducing apoptosis [19]. This could facilitate the compartmentalization of these inactivated macrophages within the alveoli and T cells in the interstitium. With the transition from an early lesion to a necrotic lesion, thereby initiating the disease process, there might be a change in macrophage morphology, their PDL and PD1 expression, and an influx of T cells into the alveoli.

This study aimed to examine this hypothesis using a immunohistopathological analysis of unique and optimally preserved untreated human autopsy material from 1931–1947.

## 2. Material and Methods

The tissue for this study was collected from pathology archives stored at the Gades Institute of Pathology, Haukeland University Hospital, Bergen, Norway, from 1931 to 1947.

Among 564 TB cases found in autopsy protocols, lung tissue in paraffin blocks was available for 81 cases. Of these 81 cases, 69 cases showed TB histology, which were further grouped into 56 cases for post-primary TB, and 13 cases for primary TB. When pneumonia was seen in the lungs, the case was grouped as post-primary TB, whereas when no pneumonia was seen in the surrounding tissue of the TB granuloma, it was grouped as primary TB [9]. 

**Figure 1:**
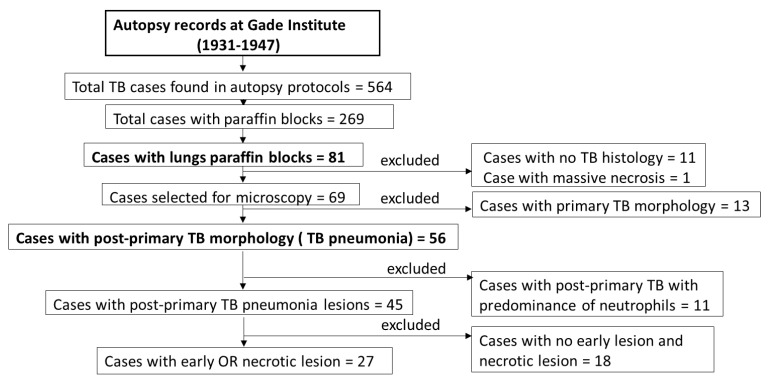
The flowchart shows the selection of cases and exclusion criteria.

Among 56 post-primary TB cases, 11 cases had a predominance of neutrophils and did not fit the morphological definition of early lesion characterized by the accumulation of foamy macrophages, and hence were excluded from this study. For the remaining 45 cases of post-primary TB, 18 did not have early or necrotic lesions and were also excluded. A total of 27 cases contained either early or necrotic lesions compatible with the post-primary TB morphology and were analyzed in this study (Figure 1).

### 2.1. Immunohistochemistry 

Immunohistochemistry targeting important immune markers (Table 1) was performed manually. After deparaffinization and rehydration, sections were boiled for 20 min in a buffer (pH 6 or pH 9) in a microwave for antigen retrieval. Tri-Buffered Saline was used to wash between each incubation step. Peroxidase block (Dako Denmark A/S, Glostrup, Denmark) was applied for 20 min to block endogenous peroxidase activity, followed by a serum-free protein block (Dako Denmark A/S) for 20 min to prevent non-specific binding. Primary antibodies (Table 1) were applied and kept for incubation at room temperature for 1 h for all markers except for in-house MTB antigens, which were kept for overnight incubation at 4 °C. The primary antibody was then followed by a secondary antibody (labeled polymer HRP anti-rabbit or anti-mouse) incubated for 40 min at room temperature. EnVision FLEX HRP Magenta Substrate Chromogen (Dako Denmark A/S, Glostrup, Denmark) was used for visualization. Antibodies for MTB antigens used in the study were in-house rabbit polyclonal. These antibodies have been used previously for immunohistochemistry analysis on human and murine lung tissues [19,20]. Proteins present in MTB cell culture are secreted antigens, while those present in cytoplasm and the cell wall are cytosolic and cell-wall-associated MTB antigens, respectively.

Tissues with TB lesions confirmed using acid-fast bacilli (AFB) were used as positive controls, and non-TB lymph nodes were used as negative controls.

### 2.2. Macrophages

Classically (M1) activated macrophages are pro-inflammatory and have a spindle-shaped morphology. They express CD80, CD86, and CD64 surface markers and secrete pro-inflammatory cytokines such as tumor necrosis factor-α, interleukin-1, interleukin-6, and inducible nitric oxide synthase. Alternatively, (M2)-activated macrophages are typically associated with tissue repair and remodeling and exhibit a more spread morphology with giant multinucleated cells. M2 macrophages express high levels of CD163, CD206, and arginase-1 [21,22].

Human monocytes and macrophages express the CD4 molecule unlike mouse macrophages [23]; therefore, CD4 T cells could not be evaluated in this study. 

### 2.3. Quantification of Immune Markers 

**Figure 2:**
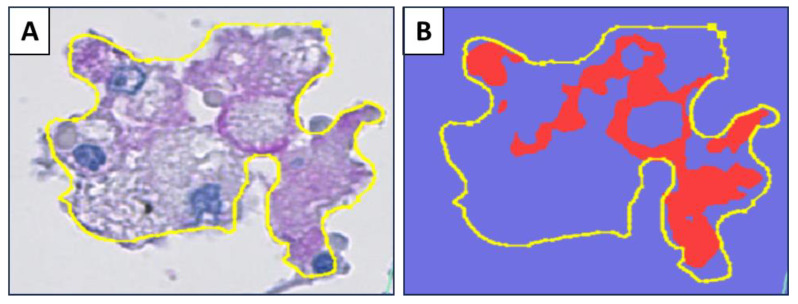
(**A**) The early alveolar lesion showing manual demarcation with a yellow line. The positive stain is magenta-colored. (**B**) Mark-up image of the lesion in A after analysis. The red area represents the positively stained area. The positively stained area divided by the total demarcated area gives the percent positive area of the lesion, which was included in the further analysis.

Stained slides for immune markers were scanned using Hamamatsu NanoZoomer XR (Iwata City, Japan) at ×40 magnification. QuPath software (0.40) was used for digital analysis [24]. Morphologically representative early alveolar lesions, necrotic alveolar lesions, and the interstitial wall enclosing these alveolar lesions were annotated manually. The region of interest analyzed was determined individually for each lesion, based on the size of the intra-alveolar accumulation of cells. The input values for stain 1 (hematoxylin), stain 2 (magenta), and the background were determined for each marker by annotating areas stained for each stain and adding the value to the image (Supplementary Figure S1A). RGB values for stain1, stain2, and background were determined for each marker separately to adjust for the staining intensity for each marker (Supplementary Table S1). The analysis was performed using a pixel classifier. The input values for the smoothing sigma and threshold value were adjusted for each marker separately according to the staining intensity of each marker (Supplementary Table S1) (Supplementary Figure S1B). The rest of the input parameters were constant for each marker. The positively stained cells were not counted individually. Quantitation of the stained cells area was performed by measuring the percentage of the area using the positively stained cells (the area shown as red in Figure 2B) in the demarcated lesion area (the area marked by the yellow line in Figure 2A,B) as follows: Positively stained area by the stain2/total annotated area × 100. 

### 2.4. Statistical Analysis

The analysis was performed using SPSS version 28.0.1. Non-parametric methods were used. Each lesion was considered as one unit, and immune markers within lesions were compared using Wilcoxon signed rank. The Mann–Whitney test was used in group analysis for cavities and cause of death.

## 3. Results

**Figure 3:**
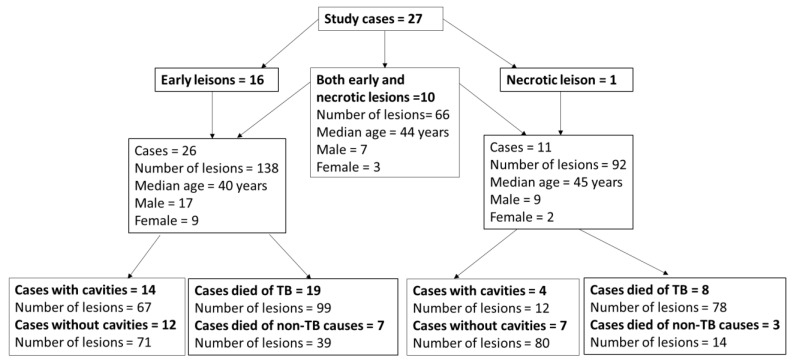
Distribution of cases and their demographics.

Among the 27 cases, there were 17 males (63%), and the median age was 42 (10–75 years). Fourteen (52%) had cavities, and 19 (70%) died because of TB. Ten of these 27 cases contained both early and necrotic lesions within the same lung, 16 cases had early lesions only, and one had necrotic lesions only (Figure 3).

**Figure 4:**
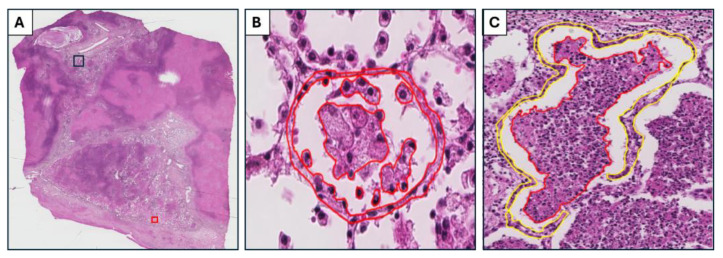
Hematoxylin and eosin-stained images of early and necrotic lesions. (**A**) The entire slide, with lesions at various stages of development identified within the same lung section. The red and black squares highlight the regions from which early and necrotic lesions were sampled, respectively. (**B**) The early lesion marked with red square in A. In the intra-alveolar lesion (demarcated with a red line), it is possible to appreciate the structure of the cells. The interstitium enclosing the alveolar lesion is demarcated with a yellow line. (**C**) The necrotic lesion is an intra-alveolar lesion in which necrosis had started, and the cell’s structure cannot be appreciated (demarcated with a red line). Interstitium enclosing the alveolar lesion is demarcated with a yellow line.

We identified the early and necrotic lesions and studied the alveolar space and corresponding interstitium as separate compartments. An early alveolar lesion was defined as an intra-alveolar lesion in which the structure of single cells could be appreciated (Figure 4A). A necrotic alveolar lesion was defined as an intra-alveolar lesion in which necrosis had started and an individual cell’s structure could not be appreciated (Figure 4B). In both lesion types, the interstitium around the alveolar lesion was defined as the interstitial wall (2–3 cell layer) enclosing the alveolar space (Figure 4A).

### 3.1. Early Lesion: Compartmentalization of Macrophages with Mixed M1/M2 Morphology in the Alveoli and T Cells in the Interstitium

**Figure 5:**
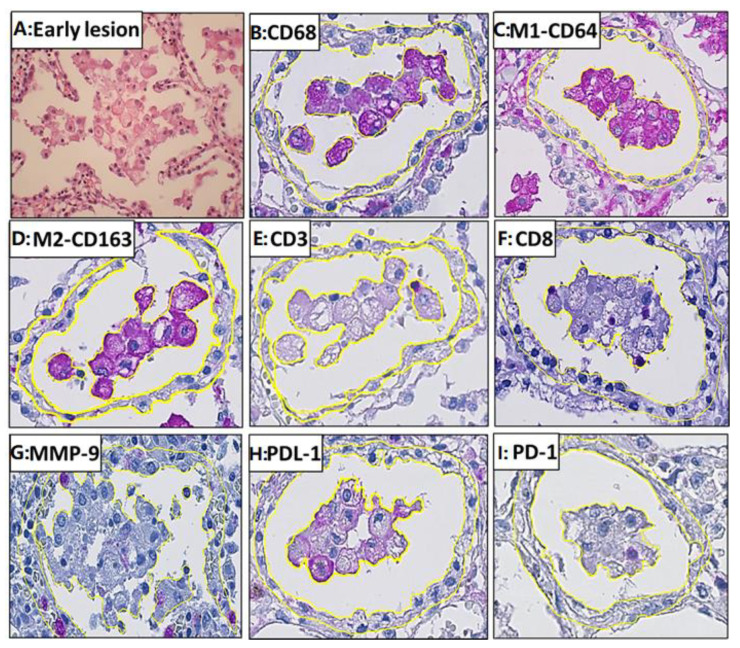
Immunohistochemistry staining of markers used in this study. (**A**) Early lesion; (**B**) CD68; (**C**) M1-CD64; (**D**) M2-CD163; (**E**) CD3; (**F**) CD8; (**G**) MMP-9; (**H**) PDL-1; (**I**) PD-1. The early lesion of post-primary tuberculosis is characterized by the accumulation of macrophages within the alveolar space. Early alveolar lesions and their corresponding interstitium are demarcated with a yellow line. The magenta color represents the positive staining and the blue color represents background hematoxylin staining.

**Figure 6:**
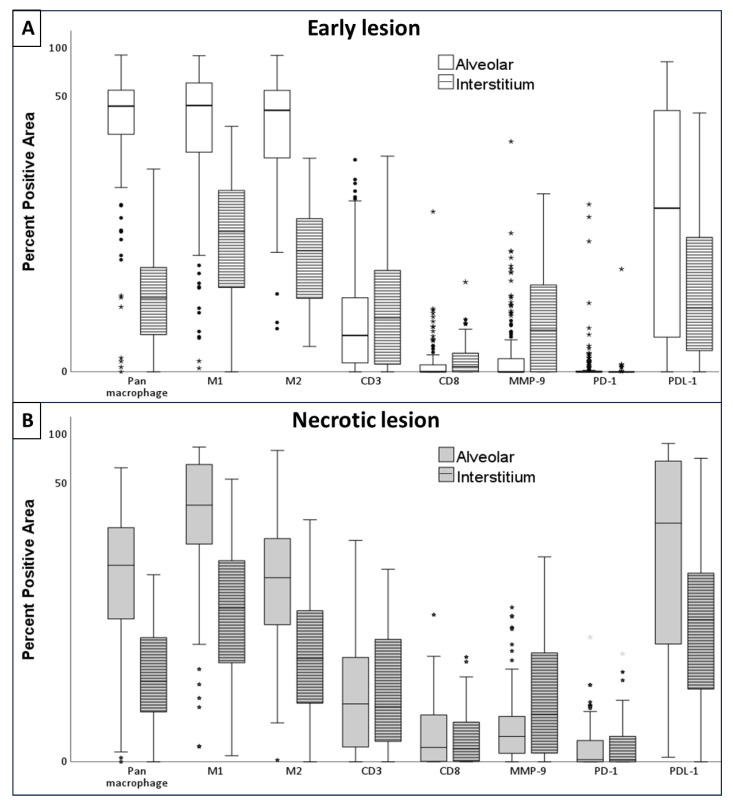
Boxplots showing the comparisons of positive percentage areas for different markers in the alveolar lesions and the corresponding interstitium. (**A**) The early lesion. (**B**) The necrotic lesion. A larger positive area for macrophages was measured in the alveolar lesion, while the interstitium had a larger positive area for T cells in both lesions.

A total of 138 early lesions from 26 patients were studied (Figure 5A). The macrophages in early alveolar lesions expressed pan macrophage (CD68), M1 (CD64), and M2 (CD163) markers (Table 2, Figure 6). All of the markers’ staining was intracytoplasmic and membrane-bound (Figure 5B–D). There was no significant difference in the positive area for the M1 as compared to the M2 macrophage markers (Figure 6A).

The staining for T-cell markers, CD3 and CD8, was membrane-bound (Figure 5E,F). Very few CD3^+^ T cells were present in the alveolar lesions, and 12% of the CD3^+^ area was CD8^+^ positive (Figure 5 and Figure 6A).

The staining for the metalloproteinase marker MMP-9 was cytoplasmic and granular. Neutrophils, alveolar pneumocytes, and monocytes were scantly expressed in the early alveolar lesions (Figure 5G). 

The staining for PDL-1 was membrane-bound as well as cytoplasmic, whereas PD-1 staining was only membrane-bound. Macrophages had PDL-1 staining, while T cells were stained by the PD-1 marker, as assessed by comparing parallel sections (Figure 5H,I).

*MTB antigens:* Both secreted, cytosolic, and cell-wall-associated MTB antigens were detected as granular intracellular staining in the cytoplasm of alveolar macrophages and monocytes (Figure 6A). MTB antigens were found in only 18% (25/138) of all the early alveolar lesions. The positive area for the BCG marker was significantly smaller than MPT46 and secreted MPT63 antigens (*p* = 0.001) (Supplementary Table S4, Supplementary Figure S2).

### 3.2. Interstitium Around Early Lesion

The interstitium around each early alveolar lesion was infiltrated by immune cells, including a mixture of macrophages and T cells. The early interstitium had a larger positive area for CD3^+^ and CD8^+^ cells compared to their alveolar lesions (Figure 6A). The CD8^+^ area constituted 7.8% of the total CD3^+^ area in the interstitium. 

Macrophages expressed pan macrophage, M1, and/or M2 markers (Figure 5). The positive area for M1 macrophages was significantly larger than M2 macrophages (*p* = 0.01) (Figure 6A). As compared to the alveolar lesion, the percent positive area for M1, M2, and PDL-1 expressing macrophages was smaller (*p* = 0.001).

MMP-9 was expressed scarcely by neutrophils, pneumocytes, and monocytes (Figure 5G). The interstitium had a larger positive area for MMP-9-expressing cells as compared to the alveolar lesions (*p* = 0.01) (Figure 6A, Table 2).

*MTB antigens:* MTB antigens were scarce in the interstitium and were found in 17% of the lesions. The distribution pattern was like those seen in the early alveolar lesions (Supplementary Figure S2). The interstitium had significantly smaller positive area for BCG, cell wall, and MPT46 antigens when compared to their respective early alveolar lesions (*p* = 0.01), whereas no difference was found for MPT63 and MPT64 (Supplementary Table S4, Supplementary Figure S2).

### 3.3. Necrotic Lesions: Influx of T Cells in the Necrotic Alveolar Lesions, Increase in CD8^+^ Cells, and Destruction of Macrophages

**Figure 7:**
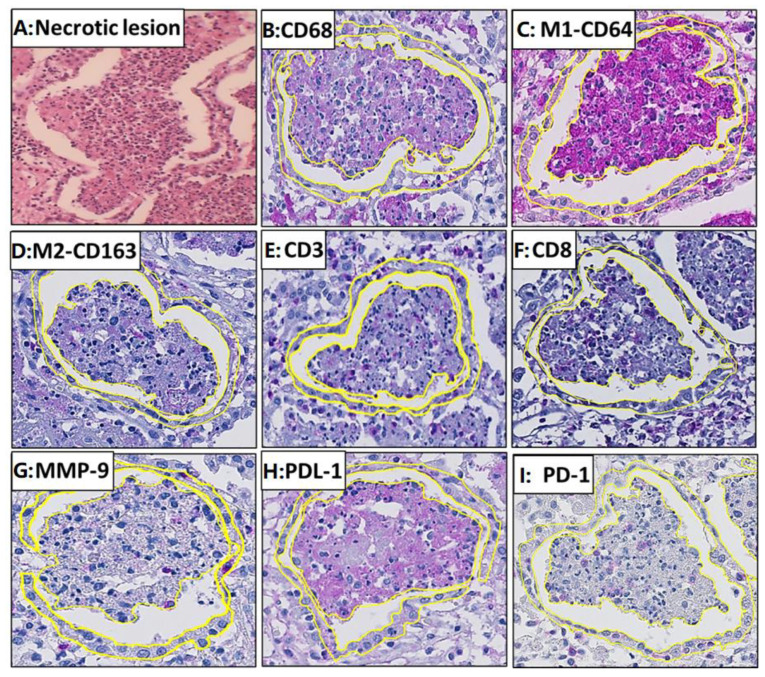
Staining with different immune markers in the necrotic lesion, and the surrounding interstitium is demarcated with a yellow line. (**A**) Necrotic lesion; (**B**) CD68; (**C**) M1-CD64; (**D**) M2-CD163; (**E**) CD3; (**F**) CD8; (**G**) MMP-9; (**H**) PDL-1; (**I**) PD-1. The magenta color represents the positive staining and the blue color represents the background hematoxylin staining. The necrotic lesion of post-primary tuberculosis is characterized with a lesion in which necrosis had started, and the cell’s structure cannot be appreciated.

A total of 92 necrotic lesions in 11 patients were studied (Figure 7A). The positive area for M1 macrophages was significantly larger than M2 macrophages (*p* = 0.01) (Figure 7B–D). CD8^+^ area constituted 27% of the total CD3^+^ area (Figure 6B and Figure 7E,F). 

*MTB antigens:* MTB antigens were found in 58% of all the necrotic lesions. The positive area for BCG and cell-wall antigens were smaller than MTP46 and secreted antigen MPT63 (Supplementary Table S2, Figure 2).

### 3.4. Interstitium Around Necrotic Lesion

The interstitium around necrotic alveolar lesions was infiltrated by immune cells constituting macrophages and lymphocytes. There was a significantly larger positive area for M1 macrophages as compared to M2 macrophages (*p* = 0.01) within the interstitium (Figure 7C,D). Positive areas for pan macrophage, M1, M2, and PDL-1 markers were significantly larger in the interstitium than in their corresponding alveolar lesion (*p* = 0.01) (Figure 7B,C,H). The positive areas for CD3^+^ cells in the alveolar lesion and interstitium were similar. The positive areas for MMP-9 were significantly larger in the interstitium compared to the necrotic alveolar lesion (*p* = 0.04) (Figure 6B and Figure 7G). The CD8^+^ area constituted 18% of the total CD3^+^ area (Figure 6B and Figure 7E,F).

*MTB antigens*: MTB antigens were found in 27% of the interstitium around the necrotic lesion in extremely low quantities in macrophages, monocytes, and neutrophils. A significantly higher percentage of positive areas was found for all MTB antigens in the alveolar lesions as compared to the interstitium (*p* = 0.01). 

The patterns of the positive areas for the different antigens were the same as seen in the necrotic alveolar lesions (Table 2, Supplementary Figure S2).

### 3.5. Comparison Between Early and Necrotic Lesions: Increase in PDL+, PD1+, and CD8^+^ Cells Associated with the Progression of Disease

A total of 10 cases with 66 early lesions and necrotic lesions, both in the same lung section, were analyzed. As early alveolar lesions progressed towards necrotic alveolar lesions there was a decrease in positive area for all macrophage markers in necrotic alveolar lesions. However, the positive areas for PDL-1, PD-1, CD8^+^, cells and MTB antigens were larger in the necrotic alveolar lesions compared to the early alveolar lesions (*p* = 0.01).

The comparison of the interstitium around early alveolar and necrotic alveolar lesions showed no change in all macrophage markers, but the interstitium around necrotic lesions had larger positive areas for PDL-1 and CD8^+^ cells (*p* = 0.04).

### 3.6. Association of MMP9 with the Formation of Cavities 

We compared the immune cells of lesions between cases with cavities and without cavities to understand the mechanism behind the formation of cavities. The positive area of MMP9 was larger in both the alveoli and interstitium of early and necrotic lesions in cases with cavities, while the macrophage markers, PDL-1, and MTB antigens covered a lesser area (Supplementary Table S2).

### 3.7. Association of Immune Cells in TB Lesions with TB Mortality 

We compared the immune cells of lesions between cases who died of TB and those who died of non-TB causes. The positive area for macrophage markers, T cell markers, PDL-1, PD-1, MMP9, and MTB antigens was larger in both the alveoli and interstitium of early and necrotic lesions in those who died because of TB as compared to those who died of non-TB causes (Supplementary Table S3).

## 4. Discussion

### 4.1. Macrophage Profile in Early and Necrotic Lesions

Macrophages are highly heterogeneous; depending on the microenvironmental stimuli, they can commit to a particular polarization status [25]. Before this study, we hypothesized that macrophage morphology in early lesions would have anti-inflammatory M2 phenotype to allow for MTB proliferation and would acquire a foamy appearance by accumulating host lipids and MTB antigens. With the transition to necrotic lesions, and thereby beginning the initiation of the disease process, macrophage morphology might change to M1. However, the results show that macrophages in early lesions did not exhibit exclusively M2 morphology. We found a mixed phenotypes of M1 and M2, whereas in the necrotic lesion, macrophages were predominately M1 phenotypes. A previous study has also shown mixed M1 and M2 macrophage morphology in the non-granulomatous lung tissues of pulmonary TB patients [26]. 

However, macrophages in early lesions expressed PDL-1, implying that these macrophages may evade immune response by exhausting T cells through the PDL1-PD1 pathway, as studied extensively in these cancers [27]. When the early alveolar lesion was compared with the surrounding interstitium, more macrophages were found in the alveoli, while more T cells were found compartmentalized in the interstitium, implying that macrophages in the early lesion may not be very active, thereby not attracting T cells in the alveoli. Earlier studies have shown that macrophages containing MTB antigens overexpress PDL-1 and the Fas ligand, which might lead to the exhaustion and/or apoptosis of T cells in alveoli, thereby creating a sanctuary for MTB within the macrophages [3,19].

### 4.2. T Cells in Early and Necrotic Lesions

It has been hypothesized that there is an influx of T cells from the interstitium to the alveolus as the lesion progresses toward necrosis. Our observations support this hypothesis. As necrosis began, CD3^+^ T cells were nearly equal in the necrotic alveolar lesion and the corresponding interstitium, in contrast to the early alveolar lesion, where they made up a larger percentage of the interstitium. Furthermore, there were more CD8^+^ cells in the necrotic alveolar lesion and the surrounding interstitium compared to the early lesion. A study performed previously on the early lesion of human post-primary TB also shows an abundance of CD8^+^ cells within the alveoli of the early lesion as well as in the interstitial walls [28], suggesting that CD8^+^ T cells play a role in disease progression and necrosis. 

### 4.3. Role of PD1-PDL-1 Pathway in TB Pathogenesis

Immune checkpoints consist of a family of receptors, including CTLA4, LAG-3, PD-1, and PDL-1. The role of the PD-1/PD-L1 signaling pathway in establishing an effective immune response to MTB infection is essential, especially in humans, as many studies have reported the reactivation of TB in cancer patients when given immune inhibition therapy [29,30]. We have studied the expression of PD-1/PD-L1 in the early and necrotic lesions of post-primary human TB. In these lesions and their surrounding interstitium, macrophages expressed PDL-1, while lymphocytes expressed PD-1. The expression of PDL-1 and PD-1 on these cells increased significantly as the lesion progressed towards necrosis. Our findings were in correspondence with a previous study performed on early lesions of post-primary TB in humans where PD-L1 expression was present in alveolar pneumocytes, sloughed bronchiolar epithelial cells, alveolar monocytes, and macrophages, and PD-1 staining was present on lymphocytes in the interstitium around the alveolar lesion [3]. Another study showed a similar staining pattern in TB granulomas in human lung tissues, showing PD-L1 highly expressed by macrophages in granulomas and PD-1 expressed on CD8^+^ T cells [31]. When PD-1 expressing cells bind it to its ligand, it activates downstream signaling pathways inhibiting T-cell activation [32]. In the present study, there was more expression of PD-1 and PDL-1 in the lesions of patients without cavities, suggesting deactivation of cytotoxic T cells and less tissue destruction. However, they were expressed more in those who died because of TB, confirming earlier findings that more expression is associated with poor prognosis [33]. The expression of PD-L1 on CD14+ monocytes and PD-1 on CD4+ T cells are significantly higher in human pulmonary TB cases and positively correlated with higher bacterial burden and worse treatment outcomes [33]. Furthermore, PD-1 expressing T cells have been shown to decrease significantly during therapy and inverse correlation with interferon γ dominant T cell response against MTB [34]. In addition, PD-1 expression is shown to increase on CD4+ T cells, producing Th1 cytokines in smear-positive TB patients compared with smear-negative patients and latently infected subjects [35]. All these findings imply the role of the PDL1-PD1 pathway in the progression of TB disease. Immune checkpoint inhibition has proven a powerful therapeutic tool in cancer patients [36], and these findings raise the possibility of its therapeutic role in TB disease, especially in extensively drug resistant TB cases with limited antibiotic options.

### 4.4. Mycobacterial Antigens

Studies performed on human lung tissues have led to the hypothesis that foamy macrophages asymptomatically accumulate MTB antigens [11,12]. When the MTB antigens reach a certain threshold, they interact with host lipids and the monolayer form of Trehalose 6,6-dimycolate (TDM), the most abundant lipid present in virulent MTB, causing tissue destruction and necrosis [37]. MTB antigens were detected in early lesions in the present study, but in low quantities, and their expression increased significantly only after the lesion started to undergo necrosis, suggesting the proliferation of bacilli as the lesion progressed. Nevertheless, the presence of MTB antigens in post-primary lesions of TB confirmed earlier observations that large amounts of antigens of MTB demonstrable by immunohistochemistry are present in the alveoli of the tuberculous lipid pneumonia [38]. Another study using the same in-house antibodies on human lung biopsy tissue showed the presence of high amounts of secreted antigens in the pulmonary lesions, which showed the morphology of pneumonia lesions of post-primary TB [19]. Viable MTB actively secretes several proteins during the growth process, which are believed to play distinct roles in TB pathogenesis. The expression of MTB antigens in the early lesions of post-primary TB and an increase in their expression with necrosis suggests that MTB use these antigens to coordinate diverse components of the host response to induce a necrotizing reaction sufficient to cause the progression of the disease [3].

### 4.5. MMP in Early and Necrotic Lesions

In pulmonary TB, MTB must drive tissue destruction to cause cavitation and dissemination to others, as pulmonary cavities harbor large bacilli populations and are the principal source of disease transmission. Moreover, cavitation is associated with treatment failure and the emergence of drug resistance [39,40]. Matrix metalloproteinases (MMPs) can degrade extracellular matrix components of lungs and play a role in cellular recruitment and tissue remodeling along with tissue destruction and cavitation in TB [41,42]. In a study, neutrophil-derived MMP-8 and -9 were associated with cavitary disease in TB patients [41]. In the present study, MMP-9 (Gelatinase B) was expressed in early and early necrotic lesions of post-primary TB; however, their expression did not seem to increase as necrosis started in the lesion. Nonetheless, they were expressed more in lesions of patients who died because of TB and patients with cavities, signifying its connection with tissue destruction and TB mortality. Studies show that MMPs play a central role in TB-associated lung injury and can be a good target for host-directed therapy to prevent tissue destruction [43]. For example, P-amino salicylic acid, a second-line drug for TB treatment, has minimal effects on TB growth in culture. However, it controls tissue destruction by inhibiting MMP-1 activity [41].

### 4.6. Immune Cell Dynamics in TB

Lung parenchyma consists of the alveolar space and interstitium infiltrated by immune cells during infection. Post-primary TB lesions show similarities to a pneumonia response, with clusters of infected macrophages contained within alveolar space and infiltrating lymphocytes within the surrounding alveolar walls. Sampling of the alveolar space of humans and non-human primates is often a proxy for the lung interstitium, which may not reflect immune cell dynamics in the interstitium as interstitium contains blood vessels and reflects systemic circulation. This limits our understanding of the early events and immune cell dynamics in post-primary TB [14]. In this study, we found differences in cellular distribution in different lung compartments depending upon the stage of the disease, thus signifying the importance of studying lung parenchyma beyond alveolar space.

### 4.7. Post-Primary TB Pneumonia with Neutrophils

Out of the 56 cases of post-primary TB, 11 were excluded due to a predominance of neutrophils in the lesions. These cases exhibited foamy macrophages containing acid-fast bacilli and MTB antigens within the alveoli, along with a high bacillary load. Earlier studies have shown that polymorphonuclear pneumonia occurs in TB, but resolves within approximately forty-eight hours, being replaced by mononuclear cells [13]. Another explanation for the presence of neutrophils in the post-primary TB lesions could be secondary infections with other bacteria. We performed polymicrobial PCR analysis on two of these cases, which tested positive for *Streptococcus pneumoniae*, *Streptococcus constellatus*, and *Haemophilus influenzae*, indicating a superinfection (unpublished data). Additionally, proteomic analysis of early lesions revealed proteins from bacterial and fungal species other than MTB, further confirming the PCR results and supporting the presence of superinfection in TB patients.

### 4.8. Strengths and Limitations

Digital image analysis has several benefits over traditional light microscopic analysis. The analysis is less subjective and reproducible, allowing for the possibility to analyze images in many ways. The most used categories of image analysis are area-based or cell-based analysis. In image analysis using Qupath software, we did area-based analysis and did not count the number of cells; the stained area was taken as a proxy for cell count. This limits direct comparisons between cells of different sizes. However, an advantage of this approach is that we could also measure areas in early necrotic lesions where the cell’s structure was not appreciable. CD4 is expressed both on human macrophages and T cells and was therefore not included, as the CD4+ T cell area could not be well classified as one cell type using digital analysis.

Not all the early lesions and necrotic lesions were selected for analysis. There was a deliberate selection bias toward lesions with clear and distinct morphologies, which warrants caution regarding the generalization of the results.

## 5. Conclusions 

Norwegian pathology archives have tissue samples available from 1931. This material is unique, as it is stored under optimal conditions along with population surveys and epidemiological registers, giving us an opportunity to relearn the pathology of human TB and address it with recent technologies to answer several of the long-standing mysteries of the pathogenesis of TB. The macrophages in the early lesions of post-primary TB expressed mixed phenotypes of M1 and M2 morphology and expressed PDL-1 and contained MTB antigens. As the lesion started to undergo necrosis, there was a more significant influx of CD8^+^ T cells expressing PD-1, and expression of PDL-1 on macrophages and MTB antigen increased, implying their role in disease progression and tissue destruction.

## Figures and Tables

**Table 1:** The immune markers used for staining.

Markers	pH for Antigen Retrieval/Dilution	Host Species	Source	Predicted Location
CD68 (Pan macrophage marker)	9/1:4000	Mouse	Dako clone KP-1	Membrane, Intracellular
CD64 (M1 marker)	9/1:6000	Mouse	Abcam OTI3D3 (Abcam, Cambridge, UK)	Membrane, Intracellular
CD163 (M2 marker)	9/1:3000	Rabbit	Abcam EPR14643-36	Secreted, Membrane, Intracellular
CD 3 (Pan T-cell marker)	9/1:100	Mouse	Dako F7.2.38	Membrane
CD 8 (Cytotoixc T-cell marker)	9/1:100	Mouse	Dako C8/144B	Membrane
MMP-9	9/1:400	Mouse	Thermo Fisher IIA5 (Greenville, NC, USA)	Cytoplasm
PD-1	9/1:400	Rabbit	Abcam EPR4877(2)	Membrane
PDL-1	9/1:200	Rabbit	Abcam SP142	Membrane, intracellular
BCG (Bacterial sonicates of BCG containing both secreted and cell-wall antigens)	6/1:4000	Rabbit	DAKO code B124; lot 063B	Cytoplasm
Polyclonal anti-body against MTB cell-wall antigens	6/1:10000	Rabbit	In-house	Cytoplasm
MPT46 (Thioredoxin Trx C, Rv3914)	6/1:1000	Rabbit	In-house	Cytoplasm
MPT63 (Secreted, Rv1926c)	6/1:1000	Rabbit	In-house	Cytoplasm
MPT64 (Secreted, Rv1980c)	6/1:1000	Rabbit	In-house	Cytoplasm

Footnotes: Programmed cell death protein 1 (PD-1), Programmed death-ligand 1 (PD-L1), Matrix metalloproteinase (MMP-9), Bacille Calmette–Guerin (BCG), *Mycobacterium tuberculosis* (MTB).

**Table 2:** The median percent and interquartile range (IQR) for the positively stained area in early and necrotic lesions.

Markers	Early Lesion n = 138		Necrotic Lesion n = 92	
	Alveolar	Interstitium	Alveolar	Interstitium
	Median (IQR)	Median (IQR)	Median (IQR)	Median (IQR)
Pan macrophage (CD68)	43.4 (28.6–54.8)	1.87 (0.69–3.44)	15.0 (6.47–26.4)	2.12 (1.00–4.87)
M1 (CD64)	43.8 (22.0–60.9)	6.41 (2.32–12.4)	36.5 (20.4–67.0)	7.77 (2.99–16.1)
M2 (CD163)	42.4 (21.0–55.4)	4.63 (1.83–7.93)	12.5 (5.79–22.8)	3.30 (1.27–7.49)
CD3	0.43 (0.07–1.29)	1.16 (0.11–3.27)	1.26 (0.23–3.40)	1.17 (0.33–4.73)
CD8	0.00 (0.00–0.10)	0.78 (0.00–0.30)	0.22 (0.00–0.97)	0.20 (0.00–0.75)
MMP-9	0.00 (0.00–0.27)	0.81 (0.00–2.46)	0.43 (0.12–0.95)	0.96 (0.13–3.66)
PD-1	0.00 (0.00–0.01)	0.00 (0.00–0.04)	0.03 (0.00–0.35)	0.26 (0.00–0.43)
PDL-1	9.34 (0.64–40.7)	1.48 (0.35–5.82)	28.1 (4.28–68.9)	6.44 (1.80–13.3)

## Data Availability

All relevant data are provided in the manuscript. Raw data can be made available upon reasonable request.

## Supplementary Materials

The following supporting information can be downloaded at: https://www.mdpi.com/article/10.3390/pathogens14030224/s1, Figure S1: A The figure shows different parameters for the image analyzed. The RGB values for stain 1, stain 2 and background were determined by making regions of interest for each stain and adding the value to the image for each marker separately. 3,3′-Diaminobenzidine (DAB) is the default name for stain2 in the software). B The figure shows the input values for pixel classifier. The smoothing sigma and threshold value were adjusted for each marker separately. The rest of the input parameters were constant for each marker; Figure S2: MTB antigens staining examples. A, C, E, G, J shows staining in early lesion and surrounding interstitium demarcated by yellow line. B, D, F, H, J shows staining in necrotic lesion and surrounding interstitium demarcated by yellow line; Table S1: Input values used for quantification. The red, green, and blue values are used for stain 1, stain 2, smoothing sigma and threshold value; Table S2: The percent median values and interquartile range for the positively stained area for immune markers in early lesion and necrotic lesion for those who had cavities as compared to those who did not have cavities; Table S3: The percent median values and interquartile range for the positively stained area for immune markers in early lesion and necrotic lesion for those who died of TB as compared to those who died of non-TB causes; Table S4: The median percent and interquartile range (IQR) for the positively stained area in early and necrotic lesions for *Mycobacterium tuberculosis* antigens.

## Abbreviations

MTB	Mycobacterium tuberculosis
MMP	Matrix metalloproteinases
M1	Classically activated macrophages
M2	Alternatively activated macrophages
PDL	programmed death-ligand
TB	tuberculosis
