# Supplementary material for: Characterization of Early Lesions of Human Post-Primary Tuberculosis and Its Progression to Necrosis Using Archival Material of the Pre-Antibiotic Era
Source: Pathogens. 2025 Feb 25;14(3):224. doi: 10.3390/pathogens14030224 (PMC11944378; doi:10.3390/pathogens14030224)
Supplement: Supplementary file 1 [file pathogens-14-00224-s001.zip › pathogens-3472552-supplementary.pdf]

Title: Characterization of early lesions of human post-primary tuberculosis and its progression to necrosis using archival material of the pre-antibiotic era, 1931-1947

List of figures

1. Supplementary Figure S1: A The figure shows different parameters for the image analyzed. The RGB values for stain 1, stain 2 and background were determined by making regions of interest for each stain and adding the value to the image for each marker separately. 3,3′-Diaminobenzidine (DAB) is the default name for stain2 in the software). B The figure shows the input values for pixel classifier. The smoothing sigma and threshold value were adjusted for each marker separately. The rest of the input parameters were constant for each marker.

2. Supplementary Figure S2: MTB antigens staining examples. A, C, E, G, J shows staining in early lesion and surrounding interstitium demarcated by yellow line. B, D, F, H, J shows staining in necrotic lesion and surrounding interstitium demarcated by yellow line.

Supplementary Figure S1: **A** The figure shows different parameters for the image analyzed. The RGB values for stain 1, stain 2 and background were determined by making regions of interest for each stain and adding the value to the image for each marker separately. (3,3'-Diaminobenzidine (DAB) is the default name for stain2 in the software). **B** The figure shows the input values for pixel classifier. The smoothing sigma and threshold value were adjusted for each marker separately. The rest of the input parameters were constant for each marker.

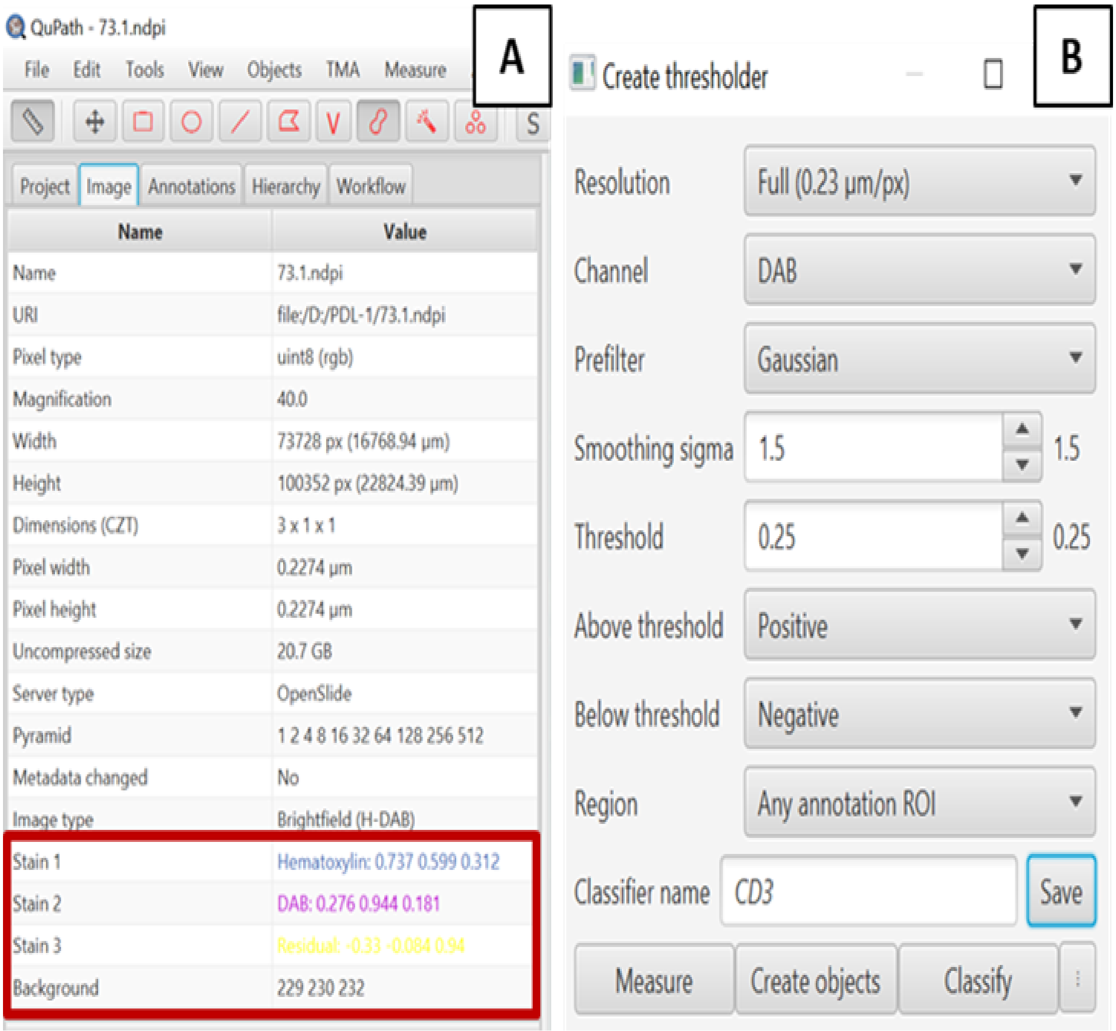

Supplementary Figure S2: MTB antigens staining examples. **A, C, E, G, J** shows staining in early lesion and surrounding interstitium demarcated by yellow line. **B, D, F, H, J** shows staining in necrotic lesion and surrounding interstitium demarcated by yellow line.

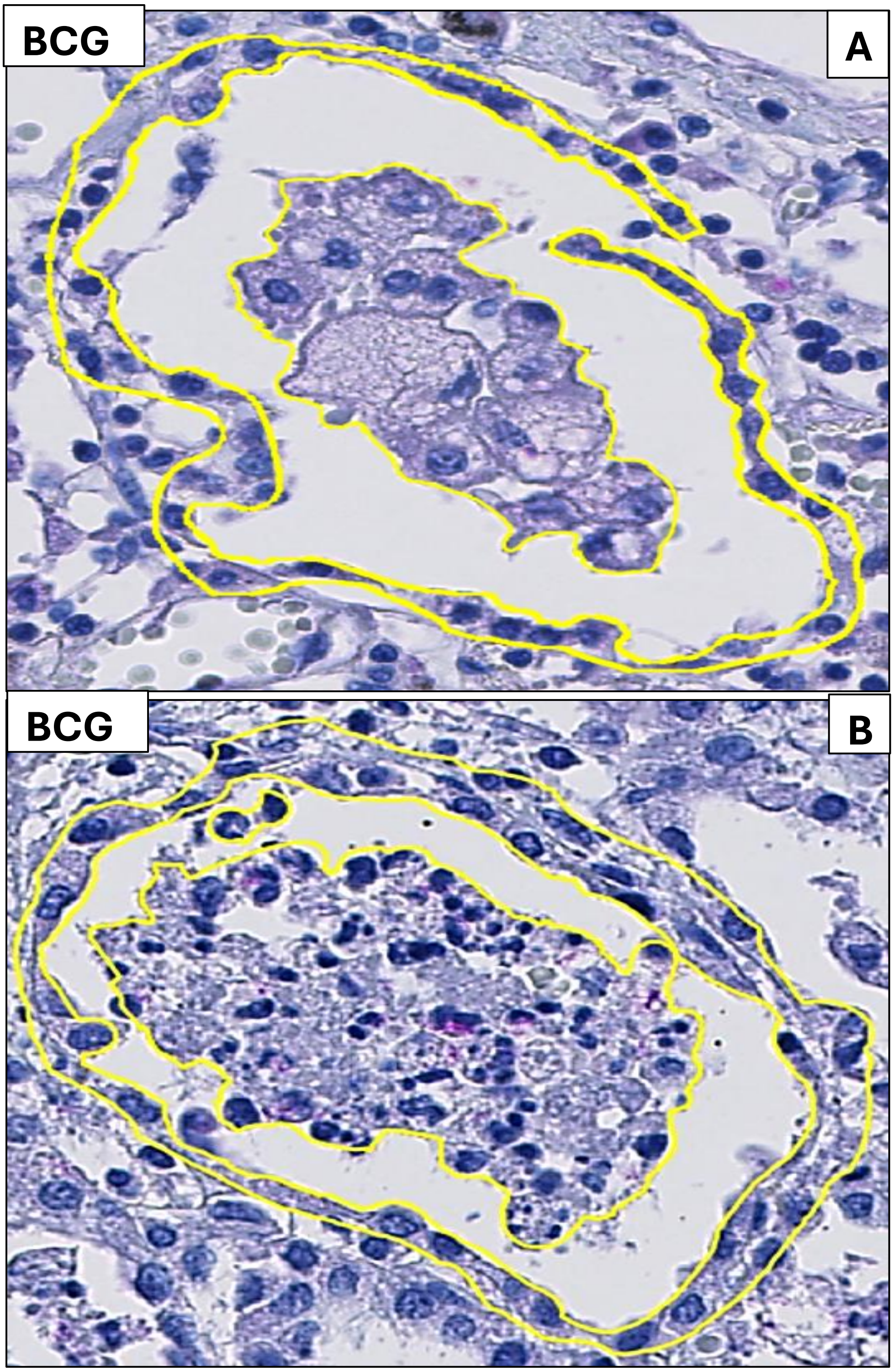

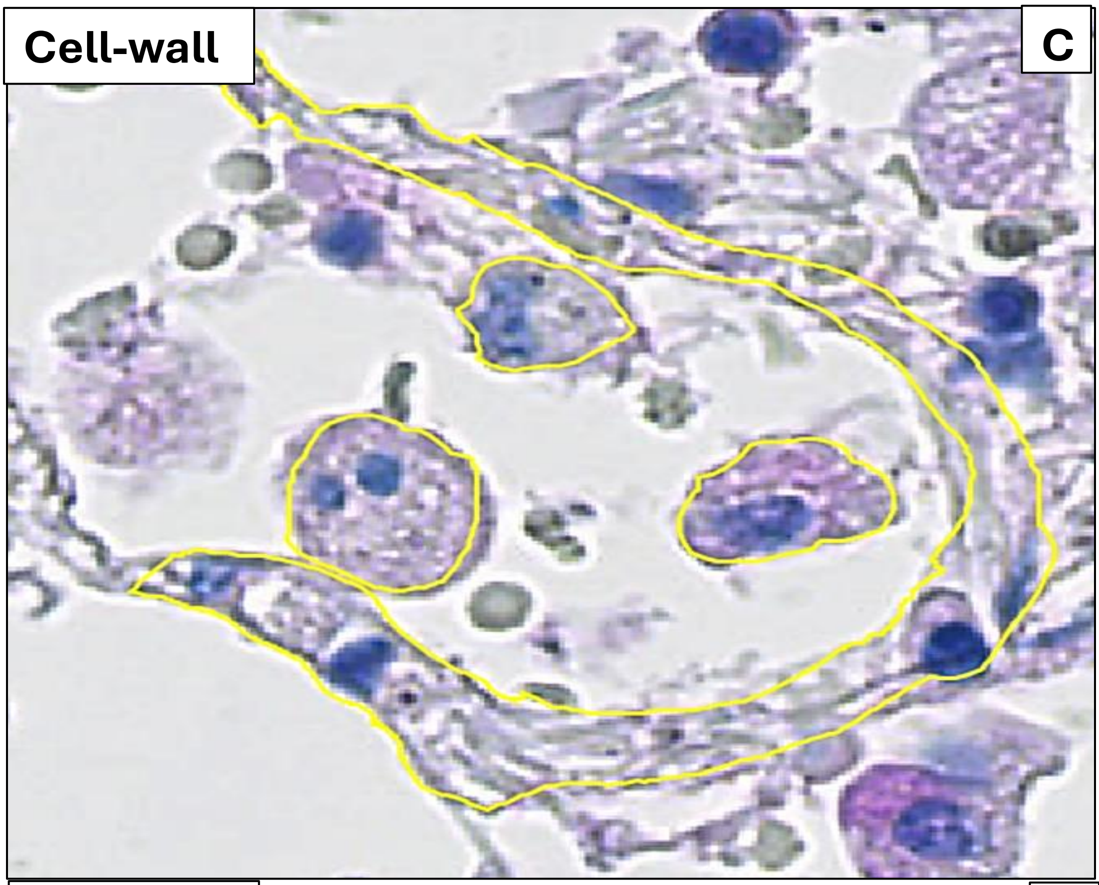
Cell-wall
C

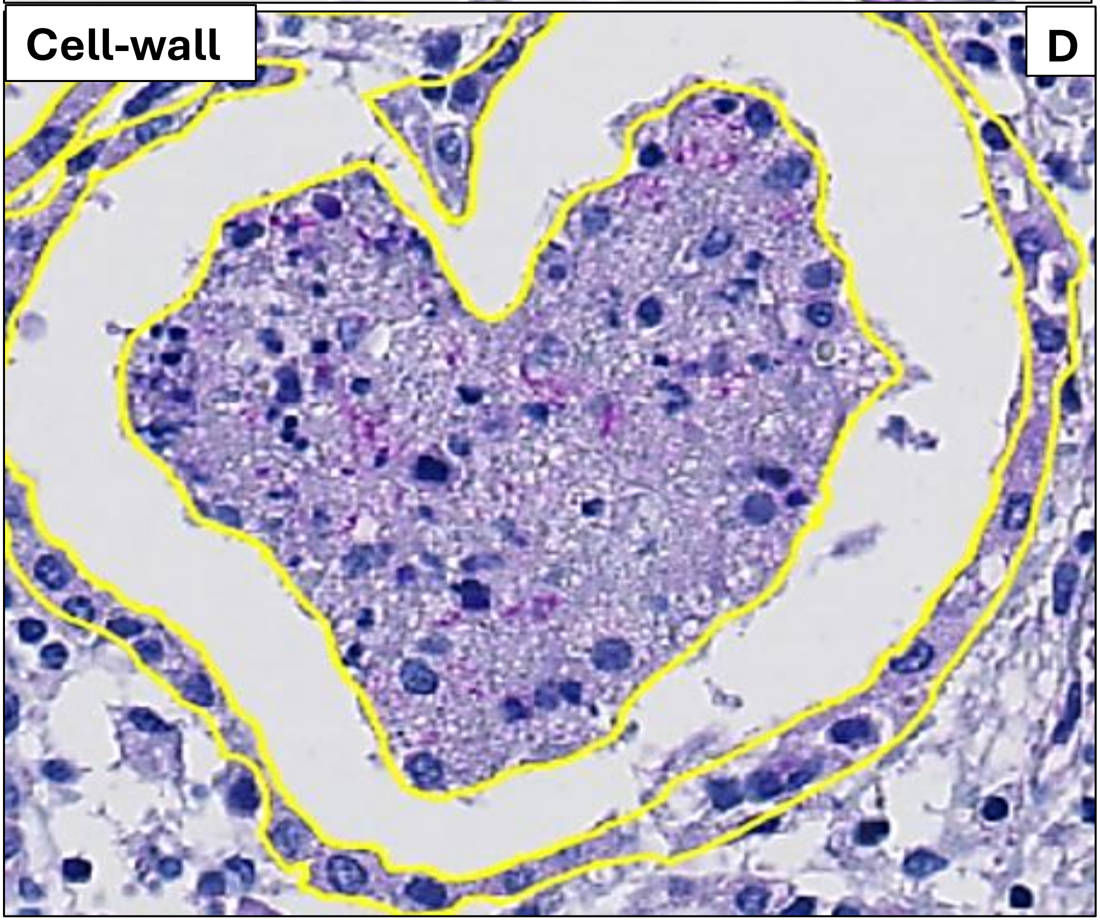
Cell-wall
D

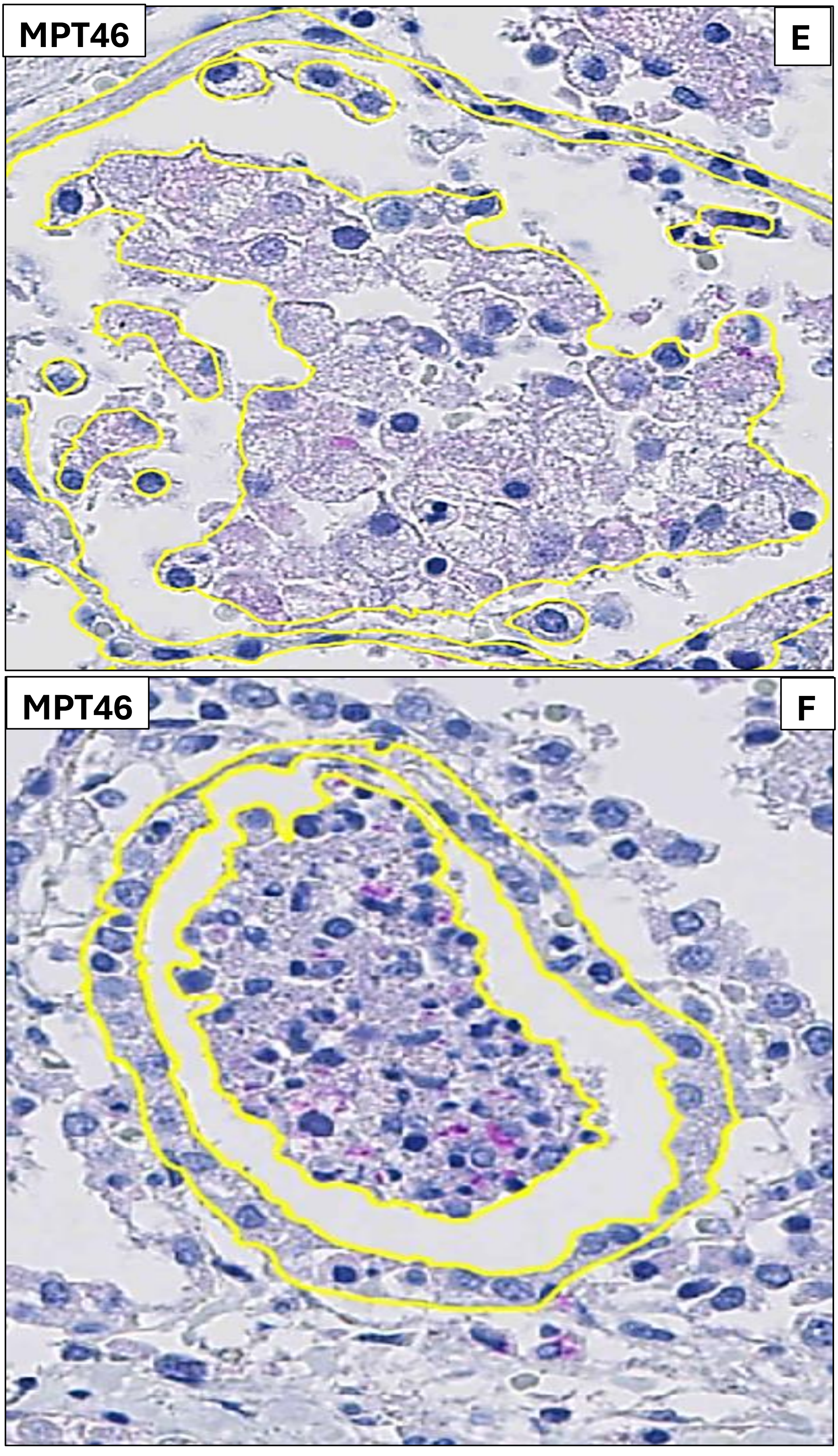

MPT46
E
MPT46
F

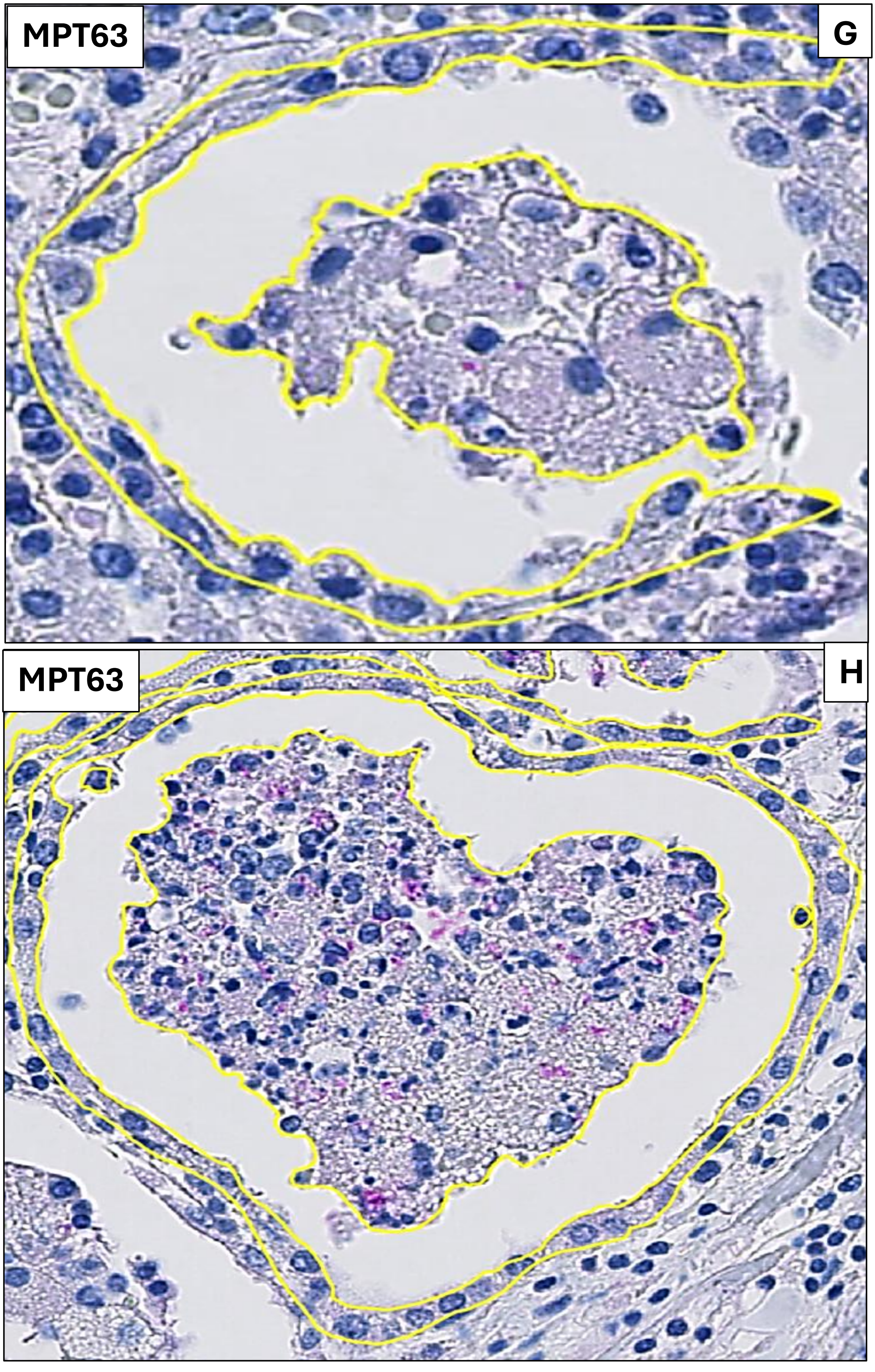
MPT63
G
MPT63
H

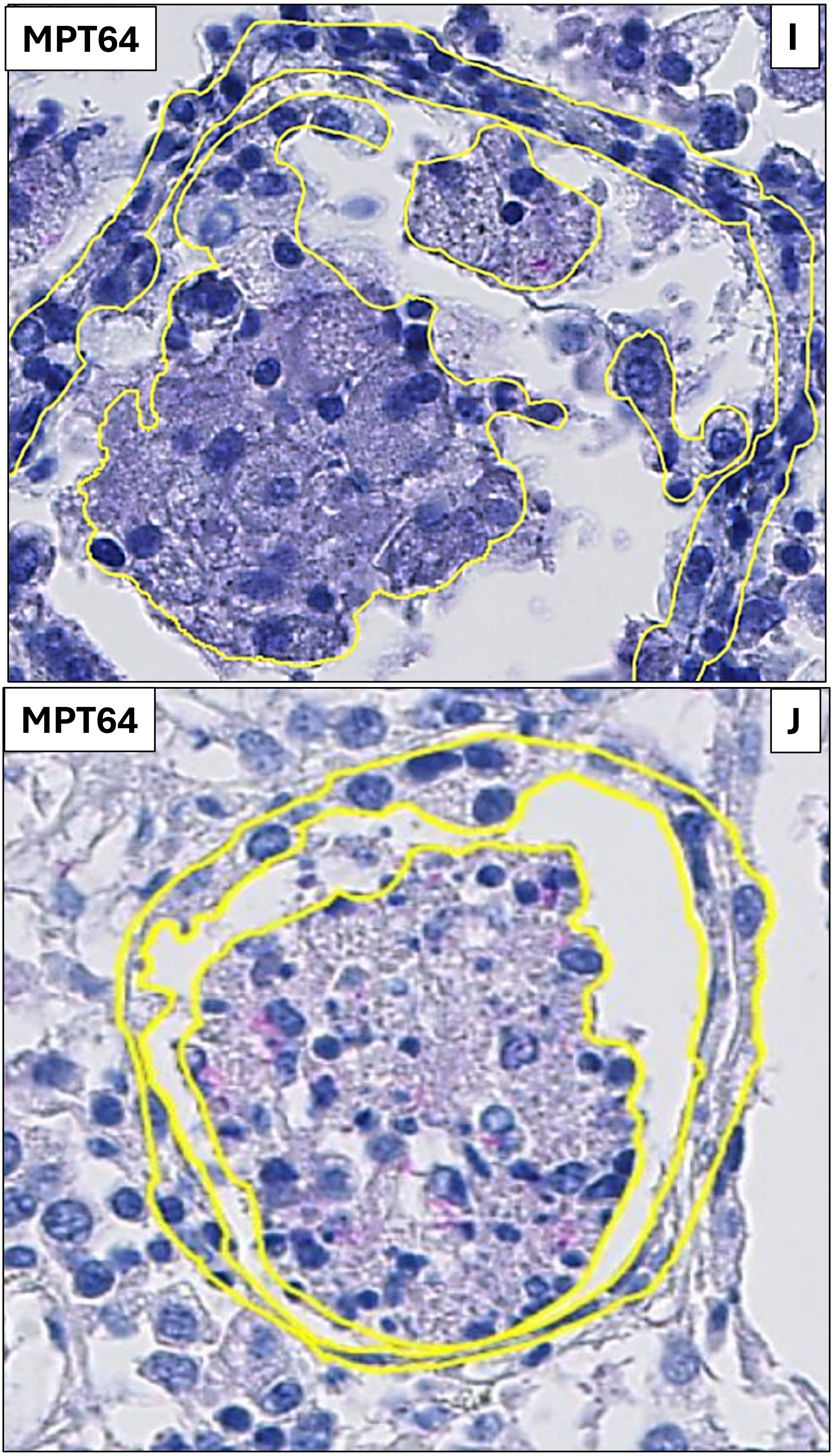
MPT64
I
MPT64
J

List of tables

1. Supplementary Table S1: Input values used for quantification. The red, green, and blue values are used for stain 1, stain 2, smoothing sigma and threshold value.

2. Supplementary Table S2: The percent median values and interquartile range for the positively stained area for immune markers in early lesion and necrotic lesion for those who had cavities as compared to those who did not have cavities.

3. Supplementary Table S3: The percent median values and interquartile range for the positively stained area for immune markers in early lesion and necrotic lesion for those who died of TB as compared to those who died of non-TB causes.

4. Supplementary Table S4: The median percent and interquartile range (IQR) for the positively stained area in early and necrotic lesions for *Mycobacterium tuberculosis* antigens

Supplementary Table S1: Input values used for quantification. The red, green, and blue values are used for stain1, stain 2, smoothing sigma and threshold value.

**Markers**	**Stain 1 RGB value (hematoxylin)**	**Stain 2 RGB value (magenta)**	**Smoothing Sigma**	**Threshold**
CD68 (Pan macrophage marker)	0.702 0.659 0.271	0.362 0.909 0.206	1.5	0.2
CD64 (M1 marker)	0.693 0.65 0.311	0.243 0.942 0.232	1.5	0.2
CD163 (M2 marker)	0.751 0.582 0.313	0.418 0.884 0.211	1.5	0.2
CD206 (M2 marker)	0.686 0.687 0.24	0.247 0.951 0.187	1.5	0.22
CD 3 (T-cell marker)	0.653 0.698 0.294	0.33 0.919 0.216	1.5	0.25
CD 8 (T-cell marker)	0.71 0.65 0.271	0.201 0.969 0.143	1.5	0.25
PD-1 (Checkpoint inhibitors)	0.727 0.622 0.291	0.299 0.94 0.163	1.5	0.12
PDL-1 (Checkpoint inhibitors)	0.737 0.599 0.312	0.276 0.944 0.181	2.5	0.1
MMP-9 (Matrix metalloproteinase)	0.159 0.975 0.156	0.763 0.604 0.23	2.5	0.12
BCG	0.274 0.941 0.198	0.651 0.701 0.29	2.5	0.15
Cell-wall	0.767 0.589 0.255	0.274 0.941 0.198	2.5	0.2
MPT46	0.736 0.6 0.313	0.274 0.941 0.198	2.5	0.2
MPT63	0.787 0.563 0.254	0.274 0.941 0.198	2.5	0.21
MPT64	0.757 0.588 0.286	0.274 0.941 0.198	2.5	0.2

Supplementary Table S2: The percent median values and interquartile range for the positively stained area for immune markers in early lesion and necrotic lesion for those who had cavities as compared to those who did not have cavities.

	**Early lesions = 26**				**Necrotic lesions = 11**			
	Cases with cavities = 14		Cases without cavities = 12		Cases with cavities = 4		Cases without cavities = 7	
	Number of lesions = 67		Number of lesions = 71		Number of lesions = 12		Number of lesions = 80	
Marker	Alveolar	Interstitium	Alveolar	Interstitium	Alveolar	Interstitium	Alveolar	Interstitium
	Median (IQR)	Median (IQR)	Median (IQR)	Median (IQR)	Median (IQR)	Median (IQR)	Median (IQR)	Median (IQR)
Pan macrophage (CD68)	44.9 (30.6-52.4)	1.39 (0.60-3.06)	40.8 (23.6-58.3)	2.24 (0.99-4.32)	4.72 (0.93-12.1)	1.47 (0.27-2.46)	14.7 (7.10-28.3)	1.91 (0.98-4.40)
M1 (CD64)	30.7 (15.0-47.3)	5.50 (1.69-9.12)	51.7 (32.2-66.8)	7.04 (2.71-13.1)	4.05 (1.23-19.8)	2.02 (0.62-6.23)	42.1 (27.2-72.1)	8.39 (3.51-17.3)
M2 (CD163)	35.7 (19.7-53.4)	3.49 (1.56-5.63)	46.9 (26.1-56.7)	5.42 (1.91-9.50)	6.13 (1.5-9.71)	1.14 (0.39-2.87)	11.4 (5.64-18.1)	3.18 (1.26-5.77)
CD3	0.31 (0.00-0.44)	0.35 (0.52-1.62)	0.92 (0.39-3.19)	2.23 (0.63-5.70)	0.97 (0.00-0.32)	0.35 (0.01-1.01)	1.70 (0.32-4.81)	2.31 (0.47-6.48)
CD8	0.00 (0.00-0.01)	0.27 (0.00-0.19)	0.56 (0.00-0.19)	0.17 (0.00-0.36)	0.00 (0.00-0.07)	0.00 (0.00-0.04)	0.46 (0.02-1.17)	0.31 (0.04-0.94)
MMP-9	0.00 (0.00-0.43)	1.24 (0.00-2.65)	0.00 (0.00-0.02)	0.46 (0.00-2.01)	2.29 (0.18-5.29)	5.29 (0.66-11.8)	0.29 (0.07-0.67)	0.92 (0.13-3.21)
PD-1	0.00 (0.00-0.00)	0.00 (0.00-0.00)	0.00 (0.00-0.15)	0.00 (0.00-0.10)	0.00 (0.00-0.00)	0.00 (0.00-0.04)	0.15 (0.01-0.52)	0.14 (0.00-0.76)
PDL-1	1.00 (0.06-23.3)	0.90 (0.24-2.77)	23.7 (5.57-46.7)	2.47 (0.56-9.25)	0.91 (0.22-3.97)	0.44 (0.13-1.11)	40.3 (9.05-70.8)	7.23 (3.95-14.2)
BCG	0.00 (0.00-0.00)	0.00 (0.00-0.00)	0.00 (0.00-0.00)	0.00 (0.00-0.00)	0.00 (0.00-0.00)	0.00 (0.00-0.00)	0.00 (0.00-0.24)	0.00 (0.00-0.00)
Cell wall	0.00 (0.00-0.00)	0.00 (0.00-0.00)	0.00 (0.00-0.00)	0.00 (0.00-0.00)	0.00 (0.00-0.17)	0.00 (0.00-0.00)	0.00 (0.00-0.35)	0.00 (0.00-0.00)
MPT46	0.00	0.00	0.00	0.00	0.00	0.00	0.00	0.00

	(0.00-0.00)	(0.00-0.00)	(0.00-0.00)	(0.00-0.00)	(0.00-0.08)	(0.00-0.01)	(0.00-0.55)	(0.00-0.00)
MPT63	0.00 (0.00-0.00)	0.00 (0.00-0.00)	0.00 (0.00-0.00)	0.00 (0.00-0.00)	0.00 (0.00-0.00)	0.00 (0.00-0.00)	0.03 (0.03-0.89)	0.00 (0.00-0.00)
MPT64	0.00 (0.00-0.00)	0.00 (0.00-0.00)	0.00 (0.00-0.00)	0.00 (0.00-0.00)	0.00 (0.00-0.00)	0.00 (0.00-0.00)	0.06 (0.06-0.87)	0.00 (0.00-0.03)

Supplementary Table S3: The percent median values and interquartile range for the positively stained area for immune markers in early lesion and necrotic lesion for those who died of TB as compared to those who died of non-TB causes.

	**Early lesions = 26**				**Necrotic lesions = 11**			
	Cases died because of TB = 19		Cases died of non-TB causes = 7		Cases died because of TB = 8		Cases died of non-TB causes = 3	
	Number of lesions = 99		Number of lesions = 39		Number of lesions = 78		Number of lesions = 14	
Marker	Alveolar	Interstitium	Alveolar	Interstitium	Alveolar	Interstitium	Alveolar	Interstitium
	Median (IQR)	Median (IQR)	Median (IQR)	Median (IQR)	Median (IQR)	Median (IQR)	Median (IQR)	Median (IQR)
Pan macrophage (CD68)	42.9 (26.0-57.5)	2.05 (0.87-4.32)	43.9 (31.1-50.6)	1.49 (0.66-3.06)	14.3 (6.58-26.1)	1.97 (0.97-4.51)	20.1 (4.87-31.2)	3.84 (1.11-6.12)
M1 (CD64)	51.2 (33.0-64.2)	7.69 (4.90-13.6)	15.0 (3.07-28.7)	1.85 (0.17-6.07)	38.6 (23.4-69.4)	8.31 (3.42-16.4)	14.8 (12.2-30.2)	4.84 (2.24-9.54)
M2 (CD163)	46.9 (31.6-60.4)	5.01 (2.27-8.15)	21.1 (15.2-44.0)	2.84 (1.14-5.70)	11.1 (5.55-24.3)	3.97 (1.26-7.80)	14.3 (9.73-19.1)	1.89 (1.22-4.33)
CD3	0.74 (0.13-1.96)	1.62 (0.34-4.64)	0.13 (0.01-0.42)	0.33 (0.52-1.40)	1.26 (0.20-4.30)	1.09 (0.33-5.04)	1.30 (0.33-2.10)	2.65 (0.19-4.87)
CD8	0.01 (0.00-0.18)	0.12 (0.00-0.36)	0.00 0.00-0.02)	0.03 (0.00-0.19)	0.31 (0.17-1.18)	0.22 (0.01-0.94)	0.10 (0.00-0.55)	0.10 (0.00-0.46)
MMP-9	0.00 (0.00-0.70)	0.63 (0.00-2.57	0.00 (0.00-0.00)	1.27 (0.31-2.32)	0.40 (0.13-0.78)	0.65 (0.11-2.46)	2.05 (0.06-5.65)	5.90 (0.18-11.3)
PD-1	0.00 (0.00-0.03)	0.00 (000-0.15)	0.00 (0.00-0.00)	0.00 (0.00-0.01)	0.32 (0.00-0.42)	0.01 (0.00-0.46)	0.02 (0.00-0.22)	0.69 (0.00-0.43)
PDL-1	21.4 (4.36-46.0)	2.44 (0.56-6.45)	0.11 (0.00-1.72)	0.52 (0.21-1.80)	36.3 (6.81-70.9)	6.78 (1.89-18.6)	3.87 (2.25-19.1)	3.50 (1.52-7.86)
BCG	0.00 (0.00-0.00)	0.00 (0.00-0.00)	0.00 (0.00-0.00)	0.00 (0.00-0.00)	0.00 (0.00-0.25)	0.00 (0.00-0.00)	0.00 (0.00-0.00)	0.00 (0.00-0.00)
Cell-wall	0.00 (0.00-0.00)	0.00 (0.00-0.00)	0.00 (0.00-0.00)	0.00 (0.00-0.00)	0.00 (0.00-0.37)	0.00 (0.00-0.00)	0.00 (0.00-0.00)	0.00 (0.00-0.00)

MPT46	0.00 (0.00-0.00)	0.00 (0.00-0.00)	0.00 (0.00-0.00)	0.00 (0.00-0.00)	0.00 (0.00-0.63)	0.00 (0.00-0.00)	0.00 (0.00-0.00)	0.00 (0.00-0.00)
MPT63	0.00 (0.00-0.00)	0.00 (0.00-0.00)	0.00 (0.00-0.00)	0.00 (0.00-0.00)	0.00 (0.00-0.97)	0.00 (0.00-0.00)	0.00 (0.00-0.00)	0.00 (0.00-0.00)
MPT64	0.00 (0.00-0.00)	0.00 (0.00-0.00)	0.00 (0.00-0.00)	0.00 (0.00-0.00)	0.00 (0.00-0.39)	0.00 (0.00-0.00)	0.36 (0.00-1.29)	0.00 (0.10-0.87)

Supplementary Table S4: The median percent and interquartile range (IQR) for the positively stained area in early and necrotic lesions for *Mycobacterium tuberculosis* antigens

Markers	**Early lesion n = 138**		**Necrotic lesion n = 92**	
	Alveolar	Interstitium	Alveolar	Interstitium
	Median (IQR)	Median (IQR)	Median (IQR)	Median (IQR)
BCG	0.00 (0.00-0.00)	0.00 (0.00-0.00)	0.00 (0.00-0.21)	0.00 (0.00-0.00)
Cell-wall	0.00 (0.00-0.00)	0.00 (0.00-0.00)	0.00 (0.00-0.29)	0.00 (0.00-0.00)
MPT46	0.00 (0.00-0.00)	0.00 (0.00-0.00)	0.00 (0.00-0.39)	0.00 (0.00-0.00)
MPT63	0.00 (0.00-0.00)	0.00 (0.00-0.00)	0.00 (0.00-0.70)	0.00 (0.00-0.00)
MPT64	0.00 (0.00-0.00)	0.00 (0.00-0.00)	0.00 (0.00-0.66)	0.00 (0.00-0.01)
